# The COVID-19 Intubation and Ventilation Pathway (CiVP); a Commentary

**Published:** 2020-03-25

**Authors:** Muhammad Akbar Baig

**Affiliations:** 1 Department of Emergency Medicine, Aga Khan University Hospital, Karachi, Pakistan

**Keywords:** COVID-19, SARS CoV-2, Novel Corona virus, Emergency, Ventilation

In wake of the current COVID-19 pandemic, which has taken the world by storm, it is imperative to protect the health and safety of physicians and staff involved in acute management of COVID-19 patients.

Numerous studies have been published, establishing evidence and opinion-based guides for emergency physicians, who are at the front line. Although many have established pathways for dealing with patient screening, testing, prognosis and disposition, I would like to discuss the management of the crashing patients in need of ventilation who we ought to be prepared for the most. 

As of now, it is essential to contain a crashing suspected/confirmed COVID-19 patient within the confines of a negative isolation chamber due to a high risk of aerosolization, with strict adherence to personal protective equipment (PPE), exclusively comprising of N95 or preferably a powered air purifying respirator (PAPR) (1). Most experienced staff should look after the patient, in order to minimize contamination to few personnel only. 

Since conventional methods of Non-invasive mechanical ventilation (CPAP/BiPAP) are inadvisable due to aerosol generation, it is suggested to secure a definitive airway with extreme precaution (2). 

Pre-oxygenation can be performed with a Bag Valve Mask device with positive end expiratory valve and a viral filter, if available. It is recommended to form a good facemask seal with both hands, while making sure not to deliver any positive breaths (2). 

Induction and relaxant medications should be administered at a maximum dose in order to prevent cough or gag reflex during the procedure (3). 

A video laryngoscope should be used so as to avoid having the operator position their face close to the patient. The most senior physician should attempt maintaining the airway, in order to maximally ensure first pass intubation success; however, in a failed airway scenario, attempts should be made to establish a surgical airway immediately. 

The endotracheal tube (ETT) should be positioned at a predetermined depth and secured properly. Avoid auscultation attempts to prevent instrument contamination, and look for bilateral chest rise or end tidal capnography waveform. If available, a viral filter should be connected to the adapter of the ETT, and another should be placed at the exhalation port of the ventilator (2). A plastic transparent sheet can be placed over the patient’s head and chest to prevent droplet spread (4). All contaminated instruments should be placed in a transparent bag for immediate disposal and/or decontamination. 

The ARDSnet (acute respiratory distress syndrome network) protocol should be followed for patient ventilation. In case of poor PaO2/FiO2(PF) ratio (<150), place the patient in prone position (5). Allow for permissive hypercapnia (pH > 7.2), if hemodynamics remain stable (6, 7). Do not give fluid boluses and maintain the patient in negative balance (8). If needed, place a central venous access line in femoral site for administering vasopressors to maintain adequate mean arterial pressure. 

Following intubation and initial ventilation, immediately proceed to transfer the patient to intensive care unit, after which, perform decontamination of the initial zone and the equipment used. Consider meticulous removal of PPE and debrief. 
